# Current Advances in Nanotechnology for the Next Generation of Sequencing (NGS)

**DOI:** 10.3390/bios13020260

**Published:** 2023-02-12

**Authors:** Angel Guillermo Bracamonte

**Affiliations:** 1 Instituto de Investigaciones en Físicoquímica de Córdoba (INFIQC), Departamento de Química Orgánica, Facultad de Ciencias Químicas, Universidad Nacional de Córdoba, Ciudad Universitaria, 5000 Córdoba, Argentina; gbracamonte@fcq.unc.edu.ar or guillermobrac@yahoo.ca; 2 Departement de Chimie et Centre d’Optique, Photonique et Laser (COPL), Université Laval, Québec, QC G1V 0A6, Canada

**Keywords:** next generation of sequencing (NGS), DNA detection, genotyping, enhanced strategies, nano-devices, nano-optics, nanochemistry, nanomaterials

## Abstract

This communication aims at discussing strategies based on developments from nanotechnology focused on the next generation of sequencing (NGS). In this regard, it should be noted that even in the advanced current situation of many techniques and methods accompanied with developments of technology, there are still existing challenges and needs focused on real samples and low concentrations of genomic materials. The approaches discussed/described adopt spectroscopical techniques and new optical setups. PCR bases are introduced to understand the role of non-covalent interactions by discussing about Nobel prizes related to genomic material detection. The review also discusses colorimetric methods, polymeric transducers, fluorescence detection methods, enhanced plasmonic techniques such as metal-enhanced fluorescence (MEF), semiconductors, and developments in metamaterials. In addition, nano-optics, challenges linked to signal transductions, and how the limitations reported in each technique could be overcome are considered in real samples. Accordingly, this study shows developments where optical active nanoplatforms generate signal detection and transduction with enhanced performances and, in many cases, enhanced signaling from single double-stranded deoxyribonucleic acid (DNA) interactions. Future perspectives on miniaturized instrumentation, chips, and devices aimed at detecting genomic material are analyzed. However, the main concept in this report derives from gained insights into nanochemistry and nano-optics. Such concepts could be incorporated into other higher-sized substrates and experimental and optical setups.

## 1. Introduction

**Figure 1:**
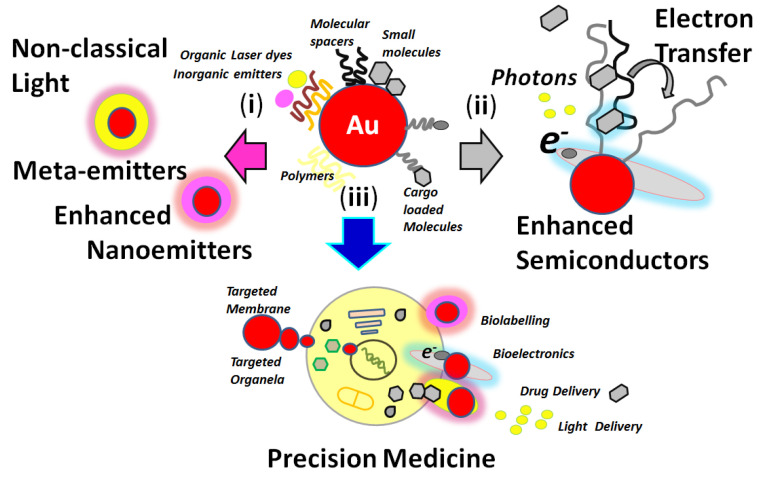
Representation of gold nanoplatforms chemical modifications and targeted studies and applications focused on: (i) nanophotonics; (ii) nanoelectronics; and (iii) nanomedicine. Reprinted with permission from ref. [10].

Over the last years, research into nanotechnology has shown to be of major importance in basic and applied science [1,2] such as in precision nanomedicine [3], nano-oncology [4,5], and smart responsive nano-optical platforms [6,7] for targeted detection of biomolecules [8] at different concentration levels. These optical active nanoplatforms could also be incorporated into substrates for devices and miniaturized instrumentation [9]. Functional nanoarchitectures could be designed with particular properties for molecular sensing. Modified metallic nanoparticles such as gold, silver, and materials such as core templates with molecular spacers with controlled dimensions could produce nano-optical platforms for chemical surface modifications (Figure 1) [10]. 

This is the case of ribonucleic acid (RNA), deoxyribonucleic acid (DNA), and related genomic materials that could interact within the near field and thus develop signal modifications recorded by nanoscopy. From these interactions, varied states of nano-aggregations could lead to new physical phenomena not yet studied as well as those already studied being potentially applied to nanotechnology and the life sciences. 

**Figure 2:**
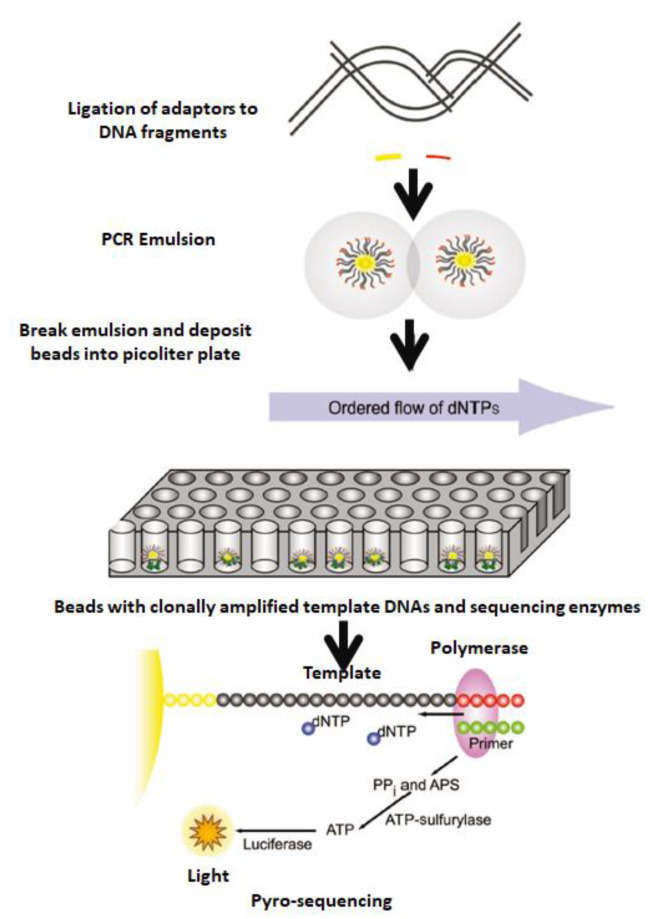
Template DNA is fragmented, end-repaired, ligated to adapters, and clonally amplified by emulsion PCR. After amplification, the beads are deposited into picotiter-plate wells with sequencing enzymes. The picotiter plate functions as a flow cell where iterative pyrosequencing is performed. A nucleotide-incorporation event results in pyrophosphate (PPi) release and well-localized luminescence. APS, adenosine 5-phosphosulfate. Reprinted with permission from ref. [15]. Copyright 2009, Oxford University Press.

In all cases mentioned, signal detection, transduction, and translation through space and time are required. In addition, the high sensitivity of optical platforms for single-molecule detections (SMD) [11] and different optical setups coupled to the molecular systems under study is well known [12,13]. Therefore, nanotechnology is a multidisciplinary research field that responds to challenges in one of the most significant research areas, namely genomic sequencing [14] and the next generation of sequencing (NGS) [15] in progress (Figure 2). In this ongoing technology, tuned nanomaterials combined with enzymes could develop new strategies for DNA extraction, separation, detection, and quantification with microbeads. Thus, the DNA library was diluted to single-molecule concentration, denatured, and hybridized to individual beads containing sequences complementary to adapt oligonucleotides. Thus, the beads were compartmentalized into water-in-oil micro-vesicles, where clonal expansion of single DNA molecules bound to the beads occurred during emulsion PCR. After amplification, the emulsion was disrupted, and the beads containing clonally amplified template DNA were enriched. 

Miniaturization of technology should be underlined to focus analysis and data recording towards individual events such as nucleotide interactions and DNA hybridization. The development of beads and nanoplatforms forming part of more complex functional machineries is applied [16,17].

It is known that sequencing addresses many challenges such as mismatching [18], low concentration in real samples, [19], signal detection after complementary DNA interaction [20], sample handling, clean-up of samples [21], and manipulation and multi-step procedures [22], all varying according to the methodology used. As could be observed, many variables should be controlled to design a new targeted methodology. This review discusses new developments from nano-optics and nanoscale control of materials with potential applications in NGS. In the first place, the basis of PCR [23], the most widely used technology in NGS [24,25], should be well understood, where nanotechnology is incorporated and, at the same time, requires new approaches based on different physical and chemical phenomena [26,27]. From these perspectives, recent nano-optic developments have been discussed that center on sequencing nanotechnology based on fluorescence, synthetic non-classical light, luminescence, and enhanced phenomena by controlling high-intense electromagnetic fields from the nanoscale [28].

## 2. Current Technologies towards the Next Generation of Sequencing

**Figure 3:**
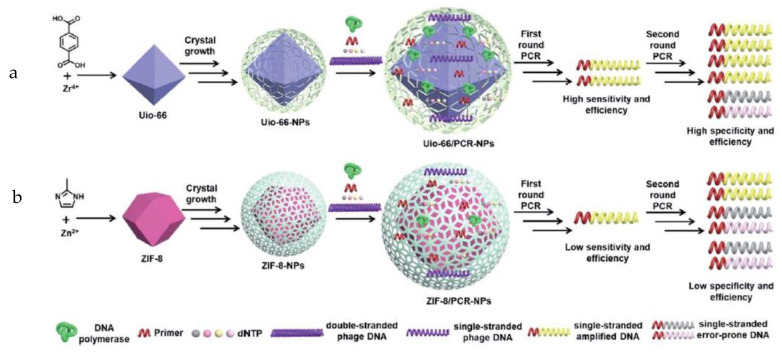
Mechanism speculation of improved PCR by UiO-66 and ZIF-8. M, DL2000 marker: (**a**) PCR was carried out with different Taq polymerases and 0.1 mg L1 l-DNA. Left lanes 1–4, conventional PCR; middle lanes 1–4, UiO-66-assisted PCR; right lane 1–7, ZIF-8-assisted PCR. The final concentrations of Taq polymerase from lane 1 to 4 are 0.25 U, 0.5 U, 1 U, and 2 U per 10 mL, respectively. In (A and C), the white frame indicates the location of the target band in all lanes. (**b**) The possible interaction among templates, Taq polymerase, and UiO-66 (or ZIF-8) during PCR. Reprinted with permission from ref. [32]. Copyright 2020, RSC.

For neophytes in this technique, the PCR method consists of a complex system based on enzymatic engineering that can read targeted DNA and incorporate complementary oligonucleotides by nucleophilic substitution [29]. From very low oligonucleotide concentrations found in real samples, concentrations may be increased to levels that could be detected and quantified by a colorimetric technique. This could be regarded as the most well-known methodology used on the market; however, it is time-consuming and produces high costs linked to the use of specific biological and chemical reagents. For these reasons, the development of modified methodologies and other derivative methods based on PCR arouses increasing interest [30]. This technique allows the provision of an important solution to detect and quantify low genomic concentrations in real samples. This is achieved by the amplification of the genomic material involving the copy of DNA by an enzymatic strategy; hence, a resulting concentration improves the signal increase in the presence of tuned nanostructures [31]. Many cycles could be repeated to control the desired quantity. However, the extra procedures add more time to the method. In addition, to improve time and procedures, other related PCR-based methods have also been developed, such as efficient polymerase chain reaction assisted by metal–organic frameworks (Figure 3) [32]. It was demonstrated that UiO-66 and ZIF-8 not only enhanced the sensitivity and efficiency of the first round of PCR but also increased the specificity and efficiency of the second round of PCR. Moreover, the modified PCR method could widen the annealing temperature range of the second round of PCR, probably due to the interaction of DNA and Taq polymerase with MOFs. A potential candidate for enhancing PCR is thus offered, yielding insights into mechanisms for improving nano-PCR and exploring a new application field for MOFs.

**Figure 4:**
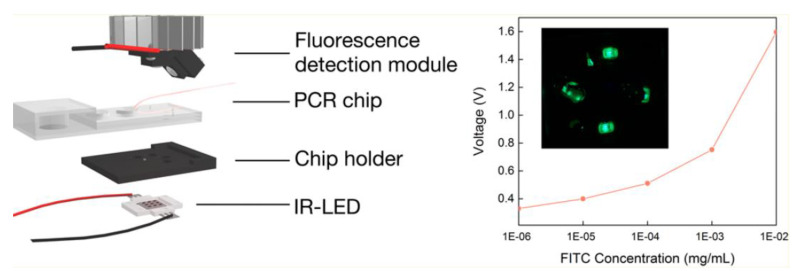
Schema of infrared-mediated RNA isothermal RT-PCR (IR-MERIT PCR) platform and its compatible multichamber microfluidic chip for simultaneous amplification and testing (SAT) detection. This microfluidic chip integrates RNA extraction, micropump, and multitarget detection functions onto the same chip. Insert Image (i) Relationship between fluorescence intensity of FITC Biolabeler at varied concentrations and applied voltage. Embedded picture is a typical laser fluorescence microscopy imaging of the labeled genomic material obtained by this methodology. Reprinted with permission from ref. [33]. Copyright 2018, American Chemical Society.

New approaches and setups have been designed by incorporating miniaturization of instrumentation and minimizing sample handling with devices and chips (Figure 4) [33]. Rapid and portable PCR detection is essential to regularly screen sexually transmitted infections. An infrared-mediated RNA isothermal RT-PCR (IR-MERIT PCR) platform and its compatible multichamber microfluidic chip have been reported for simultaneous amplification and testing (SAT) detection. This microfluidic chip integrates RNA extraction, micropump, and multitarget detection function in the same chip. Using an IR-light-emitting diode (LED) as a heat source, this platform can achieve isothermal amplification in 70 min.

**Figure 5:**
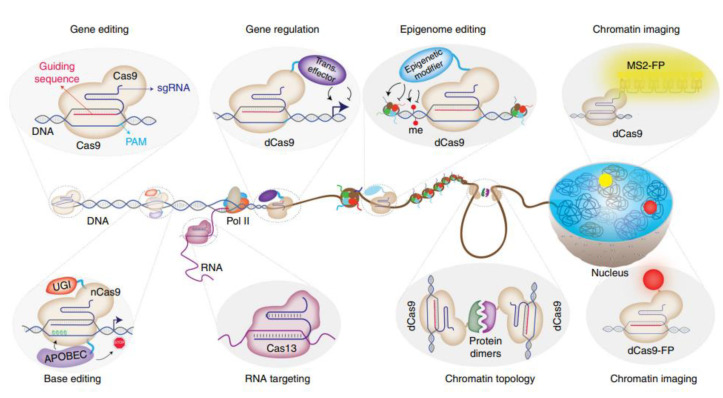
CRISPR technology: Beyond genome editing. Major application areas of CRISPR-Cas-based technologies beyond genome editing. While WT Cas9 enables genome editing through its guidable DNA cleavage activity, catalytically impaired Cas9 enzymes are repurposed to achieve targeted gene regulation, epigenome editing, chromatin imaging, and chromatin topology manipulations. Furthermore, the catalytically impaired nickase Cas9 enzyme is used as a platform for base editing without double-strand breaks. In addition to DNA-targeting Cas proteins, novel RNA-targeting CRISPR/Cas systems have been described as well. Reprinted with permission from ref. [40]. Copyright 2018, The Author(s).

With the aim of minimizing volumes, quantities, and time, confined PCR assays in microfluidic droplets on chips [34] and alternative and portable DNA amplification technologies have been developed, including loop-mediated isothermal amplification (LAMP) [35] and recombinase polymerase amplification (RPA) [36]. However, they are all based on amplifying DNA samples to generate measurable concentrations so as to be detected with advanced and standard instrumentation available in biochemical laboratories. Moreover, quantitative polymerase chain reactions (qPCR) are used to monitor relative changes in very small amounts [37]. This methodology was based on traditional PCR with the incorporation of reverse transcription of total RNA or mRNA to complementary DNA (cDNA) by applying enzyme reverse transcriptase [38], followed by quantification based on fluorescent reporters [39]. Similar techniques also include those where complex enzymatic machineries can cut, copy, and insert new, short DNA oligonucleotides in the desired targeted places [40] in long, double-stranded DNA chains [41] (Figure 5). 

This method is known as clustered regularly interspaced short palindromic repeat (CRISPR) [42], a biotechnology still being developed and discussed for human applications. The Nobel Prize in Chemistry 2020 for “the development of a method for genome editing” was awarded to two researchers using this technique: one working at Max Planck Unit for the Science of Pathogens, Germany, and the other working at the University of California, USA. [43]. Thus, by applying CRISPR/Cas9, a methodology was devised to change the DNA of animals, plants, and micro-organisms with extremely high precision. In addition, CRISPR could be adapted to targeted DNA or RNA detections by incorporating fluorescence labeling in this complex enzymatic system [44,45,46]. 

It should be noted that sequencing is based on P. Berg, W. Gilbert, and F. Sanger, who won Nobel Prizes in Chemistry in 1980 [47]. Briefly, Gilbert studied the parts of the bacterial chromosome that control the reading or transcription of the genetic information. Sanger developed a method to determine the sequence of human DNA. These bases allowed copying and quantifying oligonucleotides from modified methodologies. With these strategies, NGS technology was applied to low cell concentrations and single-cell analysis [48]. We should note the importance of incorporating the nanoscale control to develop new nanoarchitectures that interact selectively with targeted oligonucleotides and single- and double-stranded DNA. Thus, other processes involved should also be developed as well, such as extraction and fragmentation of genomic material and, according to the method used, addition of labelers to sequence, copy, and reassemble the different fragments to form a genomic sequence. Detection could hence be tuned by different techniques such a capillary electrophoresis. Accordingly, variable performances are attained with high sensitivity for different targeted real matrixes. However, note that a critical difference is that NGS sequences millions of fragments in a massively parallel system, improving speed and accuracy and reducing the cost of sequencing. 

**Figure 6:**
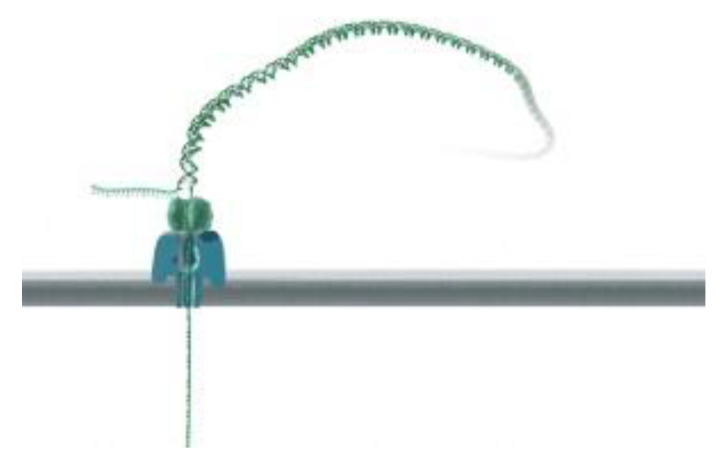
Fast track: nanopore sequencing identifies individual bases as a strand of DNA is passed through a pore. Reprinted with permission from ref. [50]. Copyright 2012 Nature News.

Moreover, the methodology was optimized to yield low quantities of real sample, and all procedures can even be automatized [49], One of the last single-molecule sequencing systems has been drastically reduced in size, namely the Oxford Nanopore Technologies’ systems by technology referred to as GridION, MinION, or Flongle. These are portable, handheld systems for RNA and DNA sequencing that read more than 2 Mb. Their detection is developed from changes in electrical conductivities from DNA strands passing through biological nanopores. Varied nucleotide sequences were thus detected (Figure 6) [50].

High-sensitivity imaging fluorescence technique allows detecting SMD in other types of systems. However, transferring this knowledge to the detection of biological molecules is also particularly required. For instance, fluorescent labeling of reversible terminators [51] was imaged on deoxynucleoside triphosphates (DNTP) to cleave them to allow adding the next base since all four reversible terminator-bound dNTPs should be present during each sequencing cycle. This method enabled an accurate human genome sequencing using reversible terminator chemistry [52]. In addition, in-flow methods could lead to handling low genomic concentrations from single-cell analysis with the right coupling of nanotechnologies such as that included in single-cell barcoding.

Fluorescence-based detection has proven to be an interesting strategy to be developed in further experimental setups. Insights gained from SMD by fluorescence-based microscopies are also of high importance in biology. The inclusion of new enhanced techniques such as metal-enhanced fluorescence (MEF) has opened up new possibilities for the development of high-sensitive sequencing. In the following sections, recent high-impact research will be discussed, underlying the control of the nanoscale and beyond.

The literature shows that the different techniques suggest versatile trends in uses and applications. Their comparison is not direct due to the type of technique required for each study. The limited availability of genomic material leads to the use of modified PCR techniques, where minimal sample handling allows amplifying the quantity. This amplified quantity could be then coupled to another technique and method. For this reason, PCR is still the most commonly known methodology used in all biochemistry laboratories. However, NGS is included in routine clinical analysis. In such a case, a new technology is available, where colorimetric detection, fluorescence signaling, labeling of genomic material and separation of sizes, and electrical signaling through substrates such as modified surfaces and porous materials could be used. Thus, performances are currently being evaluated and compared, but no study has reported on them. This opens up new possibilities for conducting studies using different NGS techniques and yielding insights into this topic.

## 3. Fluorescence Technique and Nanotechnology for Sequencing

Fluorescence is a well-known technique characterized by high sensitivity and selectivity; consequently, it is largely used for numerous purposes and particularly applied in the life sciences such as labeling of biomolecules, biostructures, and cells. Moreover, due to controlled conditions, the energy emission could be transferred, and by this manner, it could be applied as a strategy in the fluorescence resonance energy transfer (FRET) phenomena between donor/acceptor dyes [53]. Here, the signal could be tracked through time and space, producing low detection concentrations below nM in close conditions for single-molecule detection (SMD). This has allowed developing many bioanalytical techniques and bioassays. Genomic materials such as DNA with variable length and size were excellent hosts for fluorescent dye interactions, incorporations, and modifications. Numerous research works report potential applications in nanotechnology ranging from single nanoplatforms to nanoarrays and higher-sized and modified substrates for sequencing applications. In this section, a selection of recent and major developments is discussed: (i) fluorescence labeling from oligonucleotides to aptamers and longer DNA strands; (ii) incorporation of fluorescent reporters intercalated within double-stranded DNA; (iii) use of fluorescent reporters as a strategy to track DNA hybridization events; (iv) fluorescence as a strategy to couple energy transfer between fluorescent energy acceptor/donor pairs; and (v) fluorescence participating in other non-classical light pathways by varied-coupling photo-physical phenomena. As noted, fluorescence could serve as an important tool to create different detection strategies. 

**Figure 7:**
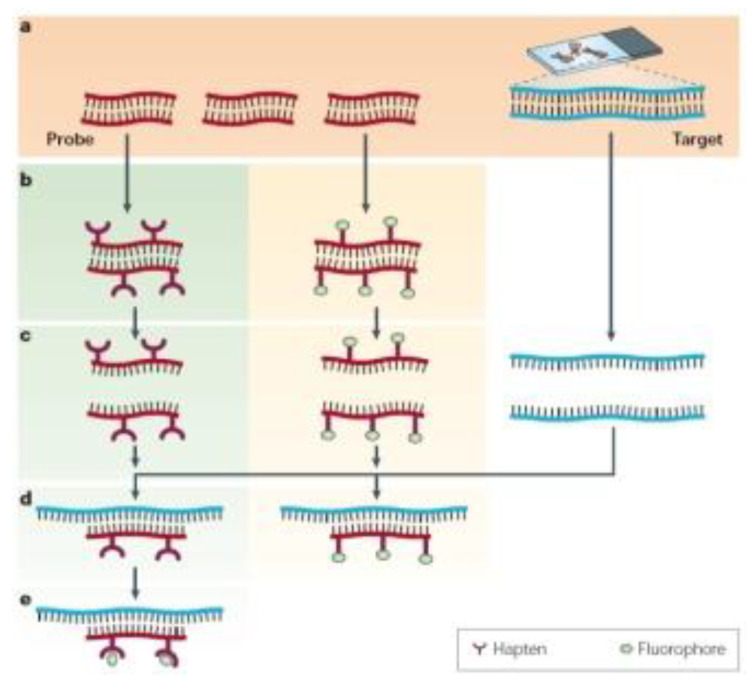
(**a**) The basic elements of FISH are a DNA probe and a target sequence. (**b**) Before hybridization, the DNA probe is labeled by various means, such as nick translation, random primed labeling, and PCR. Two labeling strategies are commonly used: indirect labeling (left panel) and direct labeling (right panel). For indirect labeling, probes are labeled with modified nucleotides that contain a hapten, whereas direct labeling uses nucleotides that have been directly modified to contain a fluorophore. (**c**) The labeled probe and the target DNA are denatured. (**d**) Combining the denatured probe and target allows the annealing of complementary DNA sequences. (**e**) If the probe has been labeled indirectly, an extra step is required for visualization of the nonfluorescent hapten that uses an enzymatic or immunological detection system. Whereas FISH is faster with directly labeled probes, indirect labeling offers the advantage of signal amplification by using several layers of antibodies, and it might therefore produce a signal that is brighter compared with background levels. Reprinted with permission from ref. [54]. Copyright 2008, Nature Education.

A very well-known fluorescence technique using fluorescent reporters such as fluorescence in situ hybridization (FISH) can be considered here. This technique has achieved the hybridization of human chromosomes by chromosome-specific fluorescent probes that label each of the 24 chromosomes with a different color (Figure 7) [54]. 

However, the detection signals recorded are still seen as an issue to improve and a challenge to overcome. Therefore, there is a particular need to conduct studies related to the transduction and amplification of the signal at a place to amplify quantities. This technique has also evolved to develop a broad range of applications and to advance DNA detection based on imaging. Similarly, fluorescent reporters to modify and label biostructures such as antibodies for DNA and RNA labeling and complementary strand targeting have also grown in importance [55]. These could be variants of the previously mentioned one; yet, the versatility of fluorescence reporters using different approaches has been demonstrated. 

Bioanalytically, a well-defined behavior is shown in the translation of this methodology to real samples. For example, DNA extraction and fluorescent complementary aptamer labeling was used to compare fluorescently labeled oligonucleotide and polynucleotide probes for the detection of pelagic marine bacteria and archaea [56]. Authors compared the detection of bacteria and archaea in the coastal North Sea and at Monterey Bay, California, after FISH either with rRNA-targeted oligonucleotide probes monolabeled with cyanin dye Cy3 (oligoFISH) or with fluorescein-labeled polyribonucleotide probes (polyFISH). In this way, different FISH-based strategies for cell counting were compared. With the best conditions achieved by using oligoFISH with monolabeled probes, authors showed detections higher than 70% of yield from the total cell counts of coastal surface waters during spring and summer.

Another example of the use of fluorescence reporters with insights into the NGS technology could be noted as follows. A multiplexed bioluminescent reporter was recently developed for sensitive and non-invasive tracking of DNA double-strand break (DSB) repair dynamics in vitro and in vivo [57]. As known, the study and tracking of DSB arouses interest due to its importance in therapeutics and treatment of diseases such as cancer. Thus, the repair of DNA damage plays a vital role in maintaining cell integrity. DBS repair is connected with two major pathways: error-prone non-homologous end joining (NHEJ) and template-dependent homology-directed repair (HDR) [58]. A study showed a non-invasive and high-sensitive bioluminescence repair reporter (BLRR) for longitudinal tracking of HDR/NHEJ both in vitro and in vivo. The method applied naturally secreted *Gaussia* luciferase (Gluc) and *Vargula* luciferase (Vluc) [59] to enable non-disruptive observation of DSB repair activities by collecting and measuring bioluminescent data from a small amount of culture medium or blood. From these tracking HDR/NHEJ events, significant differences were found in the efficiency of CRISPR/Cas9-mediated gene edition. Bioluminescent reporters to HDR/NHEJ events were thus generated within complex cellular pathways of diseases such as cancer, with repair mechanisms showing high sensitivity of CRISPR/Cas9.

**Figure 8:**
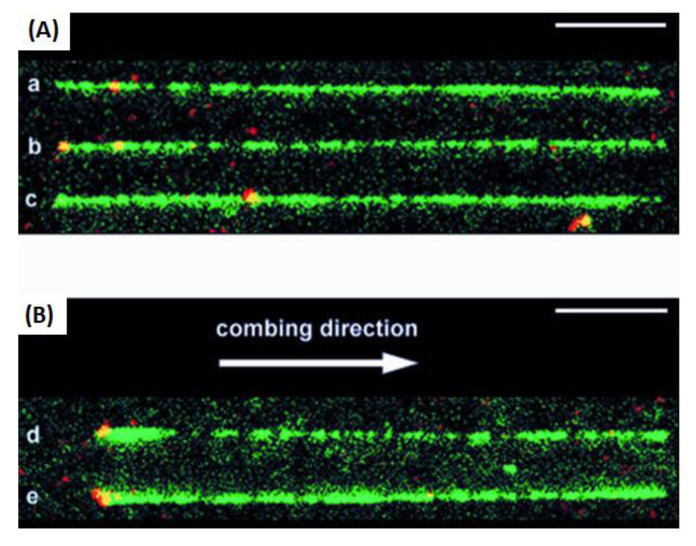
Visualization of labels on combed lambda molecules. DNA was stained with YOYO-1 (green). The labeled DNA fragments contained Alexa Fluor 546 (red); they therefore appear as yellow spots. The bars represent 5 mm. A montage of different patterns of combing and labeling was produced: (**A**) aligned longer (26 ± 27.5 mm) molecules. The combed molecules were aligned so that the red internal labels were located on the left side, but they were equally distributed on both sides with respect to the combing direction (see text). (**B**) Aligned shorter molecules (23.5 ± 25.5 mm). In this case, combed molecules were oriented with respect to the combing direction; i.e., the left side is the side where the DNA molecule sticks ^®^rst to the glass surface before being stretched in the direction indicated by the arrow. Reprinted with permission from ref. [60]. Copyright 2003, Oxford University Press.

We have shown here the generation of non-classical light in cell-tracking biological events and specific DNA interactions taking place in the different events. In all cases, complementary base interactions were not found in the detection step. In this regard, there is a huge number of techniques and methods using these particularly high-sensitive interactions. Thus, in all the cases, the detection of a signal before complementary interactions can be achieved. Fluorescent reporters as labelers of short sequences of double-stranded DNA (dsDNA) should also be considered. In this case, the strategy developed was achieved by ligating a labeled dsDNA fragment to a stem ± loop triplex forming oligonucleotide (TFO). The TFO was wound around the target sequence by ligand-induced triple helix formation; its extremities hybridized each other and left a dangling single-stranded sequence that could interact with a fluorescent dsDNA fragment using T4 DNA ligase. Hence, a non-repeated 15 bp sequence present on lambda DNA was labeled and visualized by fluorescence microscopy after DNA combing at a specific position at the targeted sequence in a long, modified linear DNA chain (Figure 8) [60]. 

Another type of labeling of double-stranded DNA involves the incorporation of small fluorescent molecules or optical reporters such as methylviologen; its implication in genome modification and related illnesses such as cancer is well known; however, the idea also allowed photoinduced electron transfer in DNA matrix from intercalated ethidium to condensed methylviologen only in the presence of all the components in a triplex conformation [61]. This strategy could be translated to other approaches in order to develop new strategies for targeted DNA strands. We can also include other types of fluorescent reporters within the IR interval of wavelengths, such as cyanine dyes. The intercalation of these molecules was studied, showing interesting properties linked to their dynamics of interaction, intercalation, groove binding, and consequent aggregation [62]. In many cases, dyes that bind to double-helical DNA by intercalation exhibit large fluorescence enhancements upon intercalation. Moreover, from simple binding of the dye as a monomer to several dyes, well-defined helical aggregates could be formed using DNA as a template. In addition, these achiral dyes are converted to chiral species within these 3D, complex structures based on the right-handed helical structure of the underlying DNA template used.

**Figure 9:**
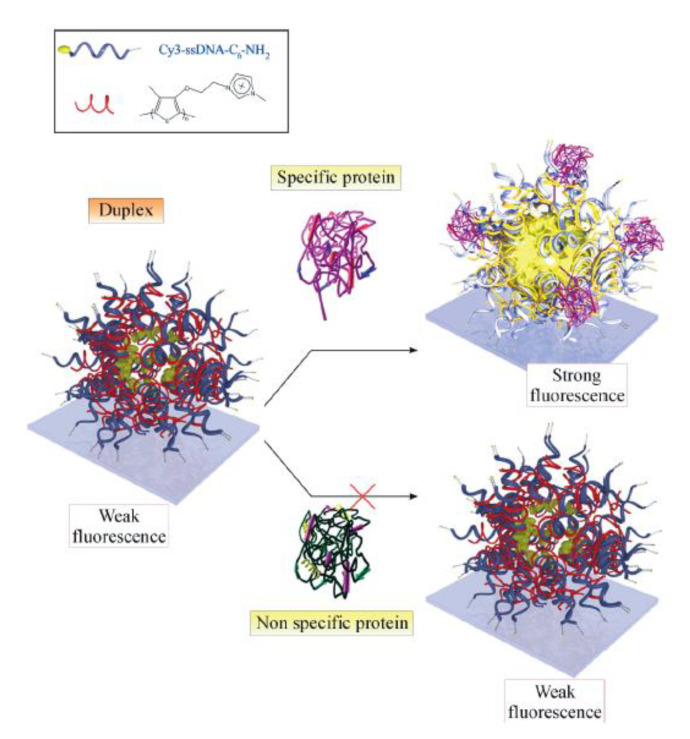
Description of the specific recognition of target proteins by single-stranded DNA (ssDNA)–polythiophene aptamer duplex aggregates on glass slides. Reprinted with permission from ref. [64]. Copyright 2006, Adv. Mater.

Other types of fluorescent reporters and intercalating agents could target short polymeric chains by non-covalent interactions. In general, these particular polymers are positively charged to interact with a negative-charge character of DNA. Cationic polythiophene could be considered as a relevant polymeric chain reported for DNA detection by different optical setups and approaches. This particular polymer showed interesting emission characteristics when interacting within the double-stranded DNA. It interacts with single strands by adopting a planar position from the bases; in the presence of the full complementary DNA strand, it changes to an anti-planar position. This triplex non-covalent complex shows an increased quantum yield compared to the duplex conformation. Many DNA aptamers-sensing systems were designed based on an amplified strategy [63], such as protein detecting arrays based on cationic polythiophene-DNA-aptamer complexes [64]. In this DNA sensor system, stoichiometric complexes (duplexes) were therefore prepared by mixing polythiophene optical transducer with a Cy3-3′-labeled ssDNA aptamer complementary target. The fluorescent reporter emission spectrum overlaps well with the absorption and emission spectra of the polythiophene, which is a necessary condition for efficient fluorescence resonance energy transfer (FRET). For specific detection of human thrombin, it was combined with cationic polythiophene sequences specific to thrombin to form stoichiometric duplexes. Therefore, from modified glass slides, fluorescent hot spots were formed in presence of the thrombin by hybridization events of labeled aptamer–polythiophene complexes only in the presence of the right target-binding sequence and protein (Figure 9). 

Thus, it significantly increased fluorescence. This strategy showed how non-covalent interactions could be tuned for specifically targeted biosensing with potential applications in DNA sequencing. We will later discuss specific examples of DNA targets and strategies from accurate functional nanoarchitectures. 

However, focusing on the implication of DNA interactions, there are other examples that show how DNA interactions could develop further strategies for DNA sensing such as innovative cases of origami architectures. We can evidence a huge origami world leading to high-accurate nanoarchitectures with non-classical tuning of light [65]. Note that only DNA strand modifications on nanoparticles could tune interactions between them in addition to forming other nanoaggregates, leading to new nano-optical properties and applications in RNA- and DNA-based strategies.

In this direction, innovative designs of non-covalent DNA assemblies at the nanoscale in the presence of cationic polythiophene have been reported. Thus, in a controlled organized media, rod micelle model-labeled duplex aggregates were formed. Massive amplification of fluorescence signal was observed upon hybridization of as few as five DNA molecules into the self-assembled structures formed. In this context, interesting optical properties were found, leading to fluorescence and light-scattering studies [66]. The fluorescence intensity of complexes is inversely proportional to scattered light intensity. Thus, duplex forms are neutrally charged, and consequently, aggregates are formed with low fluorescence emission related to the planar position of the polymer chain. On the other hand, when a labeled system with Alexa-Fluor 546 is applied as an acceptor dye to exploit a FRET pathway, it enables higher emission from the triplex structure. This was based on a switch-on mechanism of activation upon hybridization and anti-planar polymer positioning linked to an increase in the triplex emission and coupled FRET. Thus, higher quantum yield, photostability, and FRET efficiency values hinge on the conservation of an aggregated state upon hybridization. FRET measurements with a charged molecular structure similar to that of AF546 support the micellar nature of labeled aggregates, as does the observation of a slight increase in aggregate size with increasing temperature.

As observed from oligonucleotide interactions with fluorescence reporters and coupled to other photo-physical phenomena such as FRET, single-hybridization events could even be improved [67]. In addition, it is possible to apply this strategy for real-time monitoring of biochemical reactions and in vivo studies by proposing donor/acceptor pairs incorporated in different chemicals and nanostructures. Thus, innovative reading and sequencing strategies for the design of sequencers are in progress. For example, a versatile multiple-target-detection system based on DNA nano-assembled linear FRET arrays was recently reported [68]. DNA molecules are used as building blocks to create versatile nanoscale architectures and scalable and multiplex detection systems based on a FRET cascade linear DNA assembly. Thus, seven combinations of three kinds of targets were successfully detected through switch-on/off fluorescence spectra changes in the presence and absence of their respective full complementary targets. This method could be extended to a general platform for multiplex detection through FRET phenomena.

We should underline the important control required in distances between donor/acceptor emitters within more complex structures where not only well-defined architectures are involved but also other factors such as incorporation in biostructures and coupling with other components within them. For example, inter-donor-acceptor spacer length parameter was studied in RNA complex sensor structures based on dimeric modified RNA oligonucleotides with a single-molecule detection (SMD) level [69]. The Phi29 dimeric pRNA structures were used as building blocks in assembly into hexameric ring of nanomotors as modules of RNA nanoparticles. Thus, ten pRNA monomers were formed and labeled with single donor or acceptor fluorophores at various locations, and eight dimers were assembled. Single-molecule FRET signals were detected in six dimers. The tethered arm sizes of the fluorophores were estimated empirically from dual-labeled RNA/DNA standards. In this way, distances were controlled, and the hot spots were detected by fluorescence imaging when full complementary targets were achieved. In addition, it should be noted that distances between nucleotides in pRNA dimers were found to be different from those of the dimers bound to procapsid, suggesting a conformational change of the pRNA dimer upon binding to the procapsid. Interesting insights were gained during the biological interactions observed from a high-sensitive and specific fluorescence energy transfer occurring only by targeted interactions and close distances such as a few nanometers. 

In the same direction, we can include confined biostructures such as bacteria and more complex systems in vivo. Thus, a RNA aptamer was designed from a FRET sensor by positioning sequences of different sources in close proximity to single-stranded RNA origami scaffolds in the presence of targeted and pre-designed interacting fluorophores that could only be expressed in genetically encoded *Escherichia coli*. This study discussed and anticipated that the RNA apta-FRET system could have applications such as in ratiometric sensors for real-time studies in cell and be applied to synthetic biology.

In addition, the development of FRET in complex origami structures based on natural oligonucleotide interactions and modified structures has also been reported, such as in DNA origami-based FRET nanoarrays for applications such as ratiometric sensors [70]. In this DNA-based structure, distances were controlled by modifying distances between donor fluorescein (FAM) and acceptor cyanine 3 dyes with controlled and varied oligonucleotide sequences. Therefore, array brightness was optimized by calculating the FRET efficiency of the different origamis obtained. Subsequently, a ratiometric pH nanosensor was optimized using coumarin 343 as a pH-inert FRET donor and FAM as a pH-responsive acceptor. Results indicated that the sensitivity of the ratiometric sensor can be improved by arranging the dyes into a well-defined array. This concept could be transferred to new designs focused on DNA sequencing where perfect match interactions of aptamer produce bright hot spots. 

Accordingly, the accurate and controlled aggregation by highly specific and targeted DNA interactions could yield particles of varied sizes at the nanoscale and towards the microscale and higher dimensions. In this regard, recent high-tech developments have taken place in DNA sequencing that are closely related to NGS technologies offered on the market, such as nano-ball technology. This technology was initially developed from design of self-assemblies and nano-arrays, as in the case of the human genome sequencing using unchained base reads in self-assembling DNA nanoarrays [71]. Regarding to the higher sized micro-structures previously mentioned, fluorescent structural DNA nanoballs have been reported for sequencing in NGS [72]. Nanoballs are DNA self-assemblies at the nanoscale and higher scales within the microscale, with particular properties such as nucleotide transporters and bright light sources after targeted interactions. The design considers the incorporation of intercalating fluorophore in DNA strands. It could also be used as a source of nucleotides for DNA polymerization reactions, thus amplifying local concentrations of genomic materials in real time. Highly labeled DNA nanoballs functionalized with phosphate-linked nucleotide triphosphates (dNTPs) were developed as nanoplatforms of dNTPs for DNA polymerase. The particles were prepared by strand-displacement polymerization from a self-complementary circular template. Imaged by atomic force microscopy, these functionalized particles appear as condensed, fuzzy balls with diameters between 50–150 nm. They emit a bright fluorescent signal detected in 2 msec exposures with a signal-to-noise ratio of 25 when imaged using a TIR fluorescence microscope.

In order to highlight fluorescence techniques, it should be noted that fluorescence signaling in all cases showed intrinsic high-sensitive intensity. This particular property is not shown as high from non-labeled genomic materials; for this reason, it should be added in some part of the method. This addition was by using varied fluorophores, laser dyes, and emitters with different nominations depending on the current status of the development in this research field. The fluorescence signal was thus tracked after full complementary nucleotide interactions. Both steps showed to be key phenomena to detect complementary nucleotides. In view of this, the method should rely on previous information such as known genomic probe and non-classical light wavelength to measure the targeted detection, and optimally, a signal modification should be produced after genomic material detection. These three conditions could vary according to the strategy of detection, even if fluorescence is applied as a unique detection technique. Challenges posed in these three steps are connected with real-sample cleaning and experimental procedures such as chemical conjugation, labeling, and interference. Potential molecular optical active biomolecules could quench emissions and hinder oligonucleotide detections. Thus, the application of fluorescence varies by developing labeling or biolabeling with bioconjugation techniques. Associated methods such as direct fluorescence emissions, FRET, FISH, incorporation of more complex enzymatic biomachineries, as well as the development of accurate targeted aggregation have proven to be new ways to overcome difficulties in genotyping. 

To conclude this section, fluorescence techniques have been used to accomplish sequencing from the molecular level to higher-sized nanochemistry control and participate in nucleotide chemistry and DNA interaction. In this particular research field, it is very important to examine strategies already developed and transfer high-impact research in real applications to provide innovative ways to address the current challenges linked to low DNA concentrations in real samples for detection and quantification.

## 4. Enhanced Techniques and Methods for Sequencing and Genotyping

With the aim of developing new strategies for DNA detection to enhance current techniques and methods, approaches from aptamer-based point-of-care diagnostic platforms are in progress [73]. In these synthetic systems, DNA strands of varied lengths are incorporated in different bio-molecular machines where signaling is recorded after targeted interactions based on non-covalent bonding. In view of this, a broad range of well-established and novel diagnostic platforms is being considered for use in commercial point-of care (POC) diagnostics employing aptamers instead of antibodies as molecular recognition elements where light turns on the detection. In this case, an enhancement strategy is required to highlight photodetection. In this context, it could be mentioned briefly that from other developments and strategies, ideas and concepts could be taken and be transferred to new designs and developments focused on genotyping. Therefore, the grafting of surfaces and substrate modification to generate microarrays on, for instance, glass slide coating, has been largely used and demonstrated [74]. Then, this capability allowed proposing other approaches in addition to new portable technology such as selective, aptamer-based, and ultrasensitive nanogold colorimetric smartphone readouts for detection of heavy metal ions [75]. Additional functions were also incorporated as well, such as targeted bioimaging and photodynamic therapy nanoplatform using an aptamer-guided G-Quadruplex DNACarrier and near-infrared light by a selective system that delivers a photosensitizer to targeted cells and upon irradiation [76]. These developments showed improved properties not found in other developments reported. This improvement results from the incorporation of targeted components in a complex functional structure. The concept could be transferred to DNA biosensors incorporating different physical and chemical strategies and serving as a synergic methodology, enhanced pathway, and chirped laser mechanism. 

Note the tuning of fluorescence to develop new amplified signals such as biolasers and living lasers [77]. The concept is relatively easy to understand; however, generating phenomena related to broader applications proves more complex. Biolasers are generated from tune emissions of natural or synthetic dye emissions by controlling their media in biostructures such as protein complexes, where the dye is incorporated in a protective cage of the excited state. Thus, amplified signaling could produce increased and stable emission in biological media such as green fluorescent protein. This is a protein that exhibits bright green fluorescence when exposed to ultraviolet blue light [78,79]. This cage, like a stable structure, showed targeted emission wavelengths modifying their biostructure and dyes, often referred to as variants of green proteins [80,81]. Due to the high sensitivity against medium modifications, these fluorescent proteins were used as reporters of gene expression [82], and contaminants were used as heavy metal ions [83]. They were also shown to detect variation of different cell stress levels of zebrafish [84]. This small-animal model injected with the green protein was no less than twenty times more susceptible to recognizing cellular stress as compared to that not injected with this protein [85].

**Figure 10:**
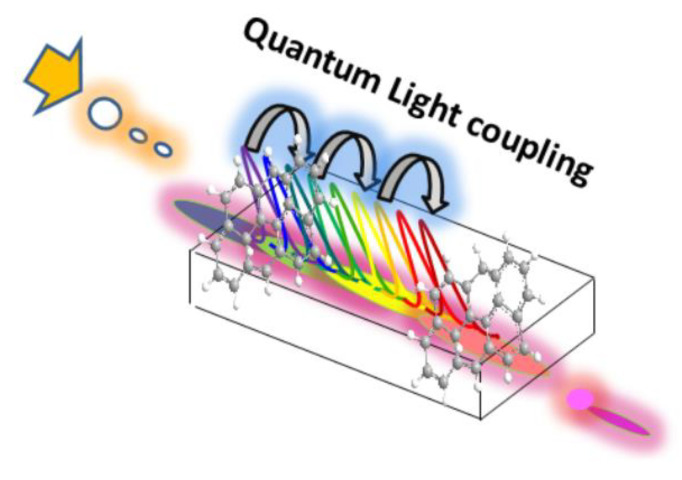
Scheme of modified substrate with optical active nanomaterials including quantum graphene dots within a metamaterial substrate for coupling and waveguiding. Reprinted with permission from ref. [88]. Copyright 2022, Current Material Science (CMS)-Recent Patents on Materials Science.

In the search of medium modifications, interactions, structures, and improved emissions, we may use targeted fluorescent polymers with the capacity to interact with single- and double-stranded DNA. For example, the fluorescence signal amplification mechanism used for direct molecular detection of nucleic acids at the Zeptomole level was largely studied and applied to different approaches and real-sample applications [86]. This improved DNA-specific detection is based on the strong interaction between the cationic polymeric optical transducer and the negatively charged nucleic acid probe that, after a targeted interaction with only a complete complementary DNA strand, activates the detection. This fact is explained by the confinement of a fluorescent DNA hybridization transducer in aggregates, improving quantum yield and photostability. Moreover, the combination of resonance energy transfer occurring in the aggregates with the use of a conjugated polymer such as hybridization transducers and donors allows ultrafast and efficient energy coupling to aggregates. The confinement and orientation of the RET donor in these supramolecular aggregates were shown to improve quantum yield and photostability, while the polymeric, structured, and conjugated nature of the DNA hybridization transducer and its proximity to the multiple acceptors makes possible an ultrafast transfer rate. Hence, the single-donor signaling can be amplified by cascade acceptor excitations. Such a report showed a procedure that can be achieved in less than 1 h with no chemical reaction. In addition, this methodology has shown to be versatile enough to detect nucleic acids of various lengths, including a segment from the RNA genome of the influenza virus. The procedure was also applied to colorimetric determinations based on the intrinsic optical activity of the duplex and triplex polymer-DNA complexes formed [87]. Here, we have shown how, from confined volumes at the nanoscale and close to the quantum scale, quantum yields could be tuned to enhanced phenomena through modified substrates (Figure 10) [88]. 

**Figure 11:**
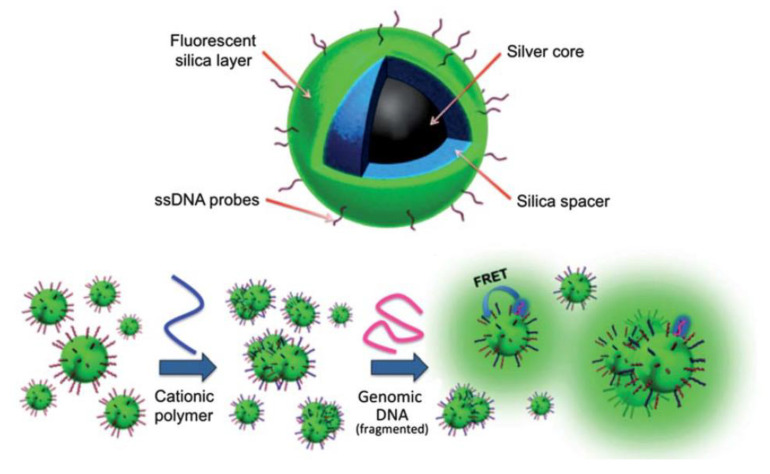
Nano-biosensor for DNA detection. Top: multi-layer core–shell nanoparticles (NPs) are made of an Ag core (35 nm) separated from an outer fluorescent silica layer by a spacer also made of silica of a few nanometers in thickness. The outer silica surface is functionalized with oligonucleotide capture probes. Bottom: schematic description of the proposed detection scheme: (1) “target-ready” nano-biosensor is prepared by mixing the cationic polymer transducer with probe-grafted NPs, and surface-charge-neutralization promotes formation of nanoparticle aggregates; (2) upon mixing the nano-biosensor with target DNA, hybridization with oligonucleotide probes occurs, which activates the polymer transducer and amplifies the optical signal via energy transfer to the fluorescent NPs. Reprinted with permission from ref. [90]. Copyright 2013, RSC.

Label-free DNA biosensing based on the accurate control of plasmonic cores and MEF could also be considered. Thus, the nanoarchitecture based on multilayer fluorescent nanocomposites was optimized using a polymeric transducer [89]. The size of silver cores, spacer lengths, and donor/acceptor spectroscopical properties was tuned. The optical active nanoplatforms were covalently linked with DNA probes to detect only full complementary DNA targets by FRET-MEF pathways. The detection events were recorded by in-flow nano-imaging. Therefore, it was applied for direct molecular detection of the SRY gene from unamplified genomic DNA (Figure 11) [90]. Further studies were developed to apply this nanoplatform and optical setups to PCR-free blood group genotyping [91].

**Figure 12:**
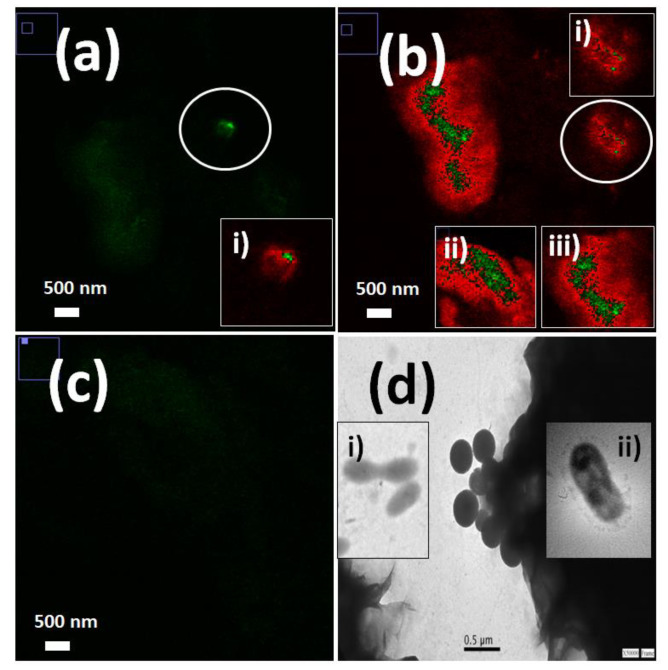
Laser fluorescence microscopy of labeled Cyanobacteria with: (**a**) mono-colored SiO2-RhB nanoparticles. Laser excitation at 543.0 nm. Inset image (i): single free SiO2-RhB nanoparticles; (**b**) mono-colored SiO2-Fl nanoparticles. Laser excitation at 488.0 nm; (**c**) multi-colored SiO2-RhB-Fl nanoparticles. Laser excitation at 488.0 nm; (**d**) TEM images of labeled Cyanobacteria with silica nanoparticles of 390.0 nm. Inset image (i) non-labeled Cyanobacteria aggregates; (ii) single-labeled Cyanobacteria. Reprinted with permission from ref. [94]. Copyright 2020, RSC.

It is important to mention the implication of new properties that could lead to meta-materials and non-classical properties where photons are modified after matter interactions [92]. In this way, nanotechnology provides many approaches based on the combination of coupled phenomena with enhanced light generation and potential bio-applications. Hybrid silica multi-colored enhanced fluorescent nanoparticles were recently developed from FRET and incorporated into two laser dyes in a confined nanoscale volume. It was thus possible to tune light emissions and intensities according to the laser excitation used [93]. This nano-emitter was applied to non-classical light delivery in unicellular microorganisms such as cyanobacteria. This led to the generation of synthetic non-classical luminescence by enhanced silica nanophotonics based on nano-bio-FRET. This effect was controlled by the laser excitation applied and energy-transfer pathway activated with optional higher and lower quantum yields according to the natural protein photo-system coupled to the biostructure (Figure 12) [94].

DNA detection for early diagnosis in real tissues is also worth mentioning. A DNA bioassay was based on a template-directed and labeled primer detected by FRET. This methodology was identified as template-directed dye-terminator incorporation (TDI) assay. Thus, it achieved the detection of mutation in the cystic fibrosis transmembrane conductance regulator (CFTR) gene, the human leukocyte antigen H (HLA-H) gene, and the receptor tyrosine kinase (RET) protooncogene associated with cystic fibrosis, hemochromatosis, and multiple endocrine neoplasia type 2, respectively. The method consisted of steps such as PCR amplification, enzymatic degradation in the excess of primers, and deoxyribonucleoside triphosphates before performing primer extension reaction. However, all these standardized steps were performed in the same tube, and the fluorescence changes were monitored in real time, providing insights into future biosensors and bioassay developments.

To understand and improve the phenomena occurring in these particular energy-transfer processes in complex matrixes with optical active biomolecules in their close surroundings, many developments have been reported in the control of the nanoscale by genomic nanomaterial formation. A case in point is the dual-channel single-molecule FRET-based dynamic DNA-detection system to establish distance parameters in RNA nanoparticles [95]. As known, FRET is highly dependent on and sensitive to the distance of the donor/acceptor emitter species. In search of genomic material imaging, systems with nanometer-scale resolution for RNA detection were studied. Hence, Phi29 dimeric pRNAs can serve as building blocks in assembly of the hexameric ring of the nanomotors, as modules of RNA nanoparticles, and as vehicles for specific therapeutic delivery to cancer or virally infected cells. In this particular complex biological system, they was calculated and used as distance parameters to optimize a known reported 3D model of the pRNA dimer. Distances between nucleotides in pRNA dimers were therefore found to be different from those of the dimers bound to procapsid. This difference results from a conformational change of the pRNA dimer upon binding the procapsid, which accounts for how biological media could differently affect energy transfer and consequent aptamer detection in further studies of genotyping applications. Fluorescent RNA aptamers could be useful as biolabelers for detecting and tracking RNA molecules into cells. One study shows a genetically encodable single-stranded RNA origami scaffold using fluorescent RNA aptamers [96]. To record a FRET-based detection signal, fluorescent aptamers were placed in close proximity to RNA scaffolds, and a new fluorophore was synthesized to increase spectral overlap. The nanoarchitecture obtained acted as an RNA device causing conformational changes in the presence of all the components, by means of which an apta-FRET signal was recorded. This phenomenon was expressed in controlled genetically engineered *E. coli*, demonstrating that the apta-FRET system was genetically encodable and that the RNA nanostructures fold correctly in bacteria. 

Moreover, DNA origami based Förster resonance energy transfer nanoarrays and their application as ratiometric sensors have also been reported [97]. In this approach, DNA acted as the main building block of an optimal targeted nanoarchitecture formation only in the presence of the full complementary DNA strand and the tunable coupled fluorescent dye incorporated. The dyes were arranged at accurate distances, where they efficiently interacted by energy transfer. In this study, the high-bright fluorescent nano-origami was applied as a pH sensor. The brightness and sensitivity of a ratiometric sensor were improved simply by arranging the dyes into a well-defined array.

In this regard, the generation of smart responsive surfaces from nanoarrays to larger modified arrays in the nanoscale control is highly required in many current technological approaches and technology centered on DNA detection and genotyping. Numerous strategies to generate signal modifications from the molecular level can be made possible by combining proper chemical surface modification by wet chemistry methods, nano-patterning, and coupling of appropriate optical setups. Therefore, targeted aptamer detection and quantification could be possible to achieve. Thus, the manipulation of low DNA concentration is the greatest challenge to overcome. The strategies to solve this issue and related ones are of high interest and impact in this field. Research into single-molecule detection (SMD) with applications in DNA targeting has been reported. A single-step FRET-based detection of femtomole DNA [98] was recently developed. This development was based on recyclable platforms of the toehold-mediated strand displacement (TMSD) process, leading to a distinct change in FRET efficiency upon target binding, which allowed a detection of a low femtomole DNA concentration without needing/requiring target amplification. The method involved manipulation of small sample sizes (fewer than three orders of magnitude compared to the typical sample size of bulk fluorescence). Furthermore, these single-molecule sensors exhibited a dynamic range of about two orders of magnitude. Thus, an evaluation of high sensitivity was carried out at the level of nucleic acid detection and identification of the single-nucleotide polymorphism (SNP), which is crucial in diagnosis of genetic diseases.

Finally, and opening the discussion of new strategies and optical setups, developments have been made by miniaturizing larger modified surfaces in microarrays, reduced-size devices, and chips. Studies have shown ultrasensitive DNA detection in microarrays by fluorescence labeling without material amplification and detection by fluorescence imaging with a single dye sensitivity [99]. With this approach, single dye molecules can be reliably detected with an average signal-to-background-noise ratio of ~42, and this result was achieved by a simple chemical modification of aldehyde surfaces. Then, fluorescence-labeled complementary oligonucleotides were hybridized at various concentrations, enabling the control of femto-molar oligonucleotide concentrations. Thus, 10 fM concentration signals of individual, specifically hybridized oligonucleotide molecules were resolved. In this way, it was shown how strategy and optical setups could be managed to provide a conceptual basis of bioassays for expression profiling of low amounts of sample material without signal amplification. Hence, there is a huge potential of DNA nanotechnology for non-classical light generation, tuning, light harvesting, signal enhancements, and biosensing developments focused on DNA detection. 

## 5. Advances and Perspectives from Nanotechnology towards NGS

The discussion of perspectives on NGS from the nanomaterial viewpoint is closely related to current nanotechnology developments. From fundamental research with new concepts and designs, proofs of concepts can be discussed and evaluated as derived from real technology. In this perspective, and knowing that NGS is focused on the determination from single nucleotides to the accurate order of variable nucleotide composition within longer DNA or RNA chains [100], we should highlighted its potential impact and capability to provide insights within other research fields.

Therefore, there are still challenges to address in relation to the main variables described as well as new ones related to further capabilities using new detection strategies and systems. In this section, we show some representative high-impact nanotechnology developments in progress, encouraging innovation in DNA-detection and -genotyping technology. 

As known, sequencing requires facing many challenges ranging from low concentrations in real samples and isolation to amplifying genomic material. The amplification procedure increases the quantity of the genomic material by copying targeted sequences. The generation of DNA-strand libraries allows continuing the design of detection strategies. Then, even if many genomic libraries are available, variable epigenetic detection could add extra difficulties. Thus, real samples by intrinsic and natural expression could affect determinations as well, and it should be contemplated in the design of the methodology. In this context, DNA mismatching should be solved. Probably, the most important variable to transduce and generate specific detection signals from single-nucleotide interactions is based on non-covalent forces. Considering natural and non-synthetic nucleotides, DNA detections from short aptamers to longer genomic chains are generally based on complementary double-stranded DNA.

Moreover, other important variables such as manipulations, clean-up, sample handling, and multi-step procedures comprising each methodological step could affect efficiency and yield, and they should be considered as well. By this manner, a multivariable system could be contemplated that could reveal additional factors on the road ahead in genetics and genomics research [101].

To find new solutions to the needs and challenges described, a multidisciplinary research field should be opened up. However, the control of the nanoscale and nanotechnology production could lead to new approaches and proofs of concepts with potential transfer to NGS technology.

Enzymatic machineries can be used to amplify genomic material such as the well-known polymerase chain reaction (PCR). For example, a rapid and efficient DNA isolation method was developed for qPCR-based detection of pathogenic and spoilage bacteria in milk [102] For neophytes, qPCR is a modified PCR-based technology that allows quantifying real-time single-oligonucleotide reading using fluorescent reporter molecules in targeted quantifications [103].

Robust technology has been developed based on natural complex enzymatic machineries such as PCR. Recently, a gene-based precision medicine technology known as clustered regularly interspaced short palindromic repeats (CRISPR) was created [104]. This biotechnology was developed to repair genomic material by incorporating new oligonucleotide sequences [105]. This is based on a complex enzymatic system that acts as an enzymatic scissor in targeted DNA sequences coupled to the replacement of genomic material [106]. Recently, two researchers working at Max Planck Unit for the Science of Pathogens, Germany, and at University of California, USA, were awarded the Nobel Prize in Chemistry 2020 for “the development of a method for genome editing” [107]. Therefore, CRISPR/Cas9 is still being studied to modify the DNA of animals, plants, and micro-organisms with extremely high precision. This technique, which is able to control fragmentation and re-incorporation of new genomic material, is particularly interesting when transferred to new strategies for DNA detection. Thus, CRISPR could generate different DNA-detection systems by adding fluorescence labelers [108,109].

From the chemical modification of nucleotides and DNA, RNA strands have provided further perspectives by integrating genomics with precision medicine. There are many relevant research studies; however, the combination of a specific nucleotide interactions accompanied by pharmacophores linking and bioconjugations for targeted drug delivery perspectives should be briefly highlighted [110]. Moreover, by this manner, non-covalent interactions are related in the targeted function.

By exploiting the concept of non-classical light from the molecular level by fluorescence labeling such as DNA-intercalating agents or by covalent bond modifications, the research work for new imaging-based developments should be continued. Recently, research has shown an integrated imaging and computational strategy to model gene folding with nucleosome resolution [111]. It was thus possible to identify a specific distribution of nucleosomes within specific genes in super resolution through the simultaneous visualization of DNA and histones. This method advanced information on chromatin accessibility for regulatory factors such as RNA polymerase II. Intercellular variability, transcriptional-dependent gene conformation, and folding of housekeeping and pluripotency-related genes were studied in human pluripotent and differentiated cells, gathering accurate data.

**Figure 13:**
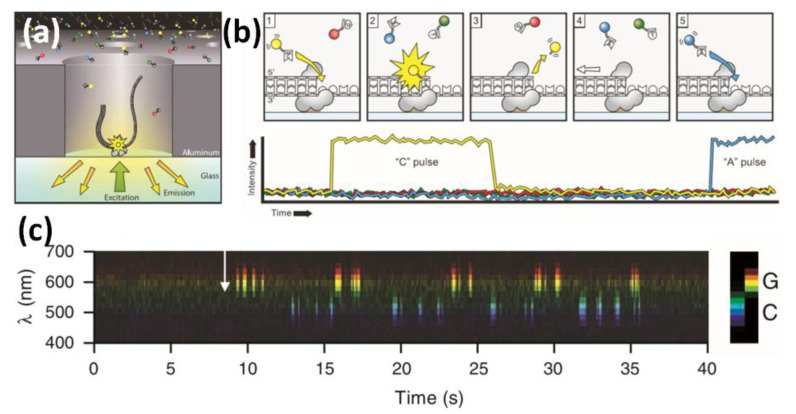
Principle of single-molecule, real-time DNA sequencing. (**a**) Experimental geometry. A single molecule of DNA template-bound F29 DNA polymerase is immobilized at the bottom of a ZMW, illuminated from below by laser light. The ZMW nanostructure provides excitation confinement in the zeptoliter (10−21 L) regime, enabling detection of individual phosphor-linked nucleotide substrates against the bulk solution background as they are incorporated into the DNA strand by the polymerase. (**b**) Schematic event sequence of the phosphor-linked dNTP incorporation cycle, with a corresponding expected time trace of detected fluorescence intensity from the ZMW. (1) A phosphor-linked nucleotide forms a cognate association with the template in the polymerase active site, (2) causing an elevation of the fluorescence output on the corresponding color channel. (3) Phosphodiester bond formation liberates the dye-linker-pyrophosphate product, which diffuses out of the ZMW, thus ending the fluorescence pulse. (4) The polymerase translocates to the next position, and (5) the next cognate nucleotide binds the active site, beginning the subsequent pulse. (**c**) Time-resolved fluorescence intensity spectrum from a ZMW. Data from a 15 × 5 pixel area from each movie frame were spatially collapsed to a 15-pixel spectrum, shown as a function of time. The arrow denotes addition of the catalytic metal ion that initiated the polymerization reaction. Reprinted with permission from ref. [115]. Copyright 2009, Science.

To develop further resolution to nano-biostructures, the molecular level, from the bottom-up design that could provide targeted biomolecular detection from proteins to amino acids and small molecules, should be evaluated. Similarly, the resolution of single nucleotides could prove a major development; yet, this level of resolution is only achieved by a highly smart responsive strategy. In this context, biophotonic strategies for single-molecule detection (SMD) level should be considered [112]. Therefore, there is a growing body of literature on DNA detection and amplification based on different optical approaches and controlled DNA grafting of surfaces [113]. For single-molecule sequencing, we may refer to the sequencing and tracking of individual nucleotides based on templated DNA exposed to a solution containing DNA polymerase and a fluorescent nucleotide. If a nucleotide were incorporated, it would be achieved by a complementary strand of the template. Thus, the fluorescence would be read using a total internal reflection fluorescence (TIRF)-based helioscope, recording the positions where the DNA strand had incorporated a fluorescent nucleotide from the solution [114]. It should also be noted that real-time DNA sequencing from single polymerase molecules [115] enabled tracking of single-nucleotide incorporation in real time by fluorescence imaging (Figure 13). The level of developments achieved in the single-molecule dynamic detection of chemical reactions [116] based on an electrochemical device is also worth noting.

Previous developments anticipated the beginning of many challenges to overcome, where biostructure and enzymes coupled to fluorescence labeling joined aptamer interactions with targeted recognitions of specific sequences, or single nucleotides were joined to optimize genomic detection. Results showed high performance; however, the key variable of low genetic material in real samples was managed by amplification. This methodology is linked to a costly and time-consuming process. Therefore, PCR-free strategies are still being developed, specifically in NGS technology.

Nanoscale control has led to new strategies to overcome low DNA concentrations. This is due to many reasons, mainly: (i) proximity of the nanoscale to the molecular level; (ii) molecular hinderance; (iii) interactions associated with non-covalent binding and physical phenomena; and (iv) amplification of the signal such as laser-based materials by different enhanced physical and chemical pathways under study. 

**Figure 14:**
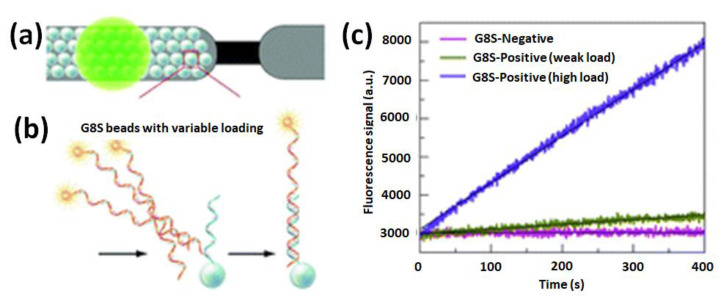
(**a**) Schematic microfluidic microarray hybridization on tightly packed monolayer bead bed inside a microfluidic cartridge for the detection of single-nucleotide polymorphisms; (**b**) real-time monitoring of DNA hybridization on spherical particles functionalized with oligonucleotide capture probes nominated as G8S. (**c**) Fluorescence emission detection from real-time monitoring of DNA hybridization events vs. the pass of time. Reprinted with permission ref. [118]. Copyright 2021, RSC.

A PCR-free DNA-detection strategy was developed from modified magnetic beads with short, targeted DNA aptamers incorporated in microelectromagnetic traps [117]. This enabled the interaction of targeted and complementary DNA strands using a fluorescent polymeric transducer in a miniaturized device that leaded to the separation of the genomic material and detection by laser fluorescence microscopy. Thus, a detection limit of ∼200 target copies in a probed volume of 150 µL (1.4 copies/µL) was obtained for a DNA sequence specific to *Candida albicans*. We should note that this detection strategy does not require the release of the hybridized target DNA prior to its detection, labeling, or amplification of nucleic acids. The preconcentration and detection steps could be achieved simultaneously on the same support. The design could be extended to the application of this ultrasensitive biosensor to biological samples with complex matrixes and to integrated lab-on-a-chip platforms, where faster multitarget DNA detections could be developed in point-of-care diagnostics and field analysis. The application of in-flow methodologies could also provide other ways of addressing this challenge such as in real-time monitoring of bead-based DNA hybridization in a microfluidic system and the study of amplicon hybridization behavior on solid supports (Figure 14) [118]. 

This development and device-based technology used for fundamental and applied research has provided interesting insights. For instance, the length of the overhanging (dangling) end of target DNA strands following hybridization to the capture probe was correlated with interactions with the complementary strand in solution, which could result in the unbinding of the target and its release from the surface. Thus, an instrument for real-time monitoring of DNA hybridization was created on spherical particles functionalized with oligonucleotide capture probes and arranged in the form of a tightly packed monolayer bead bed inside a microfluidic cartridge. This approach allowed further elucidation of the dynamics of interaction. Thus, it was known that by increasing the length of the dangling end, desorption of target amplicons from bead-bound capture probes is produced at a rate approaching that of the initial hybridization process. This led to the optimization of conditions in order to perform hybridization events from *Streptococcus agalactiae* cfb gene amplicons obtained from randomized clinical samples. The methodology presented was also used for further investigation of competitive hybridization mechanisms on solid supports to evaluate suitability, such as microarray capture probes. Similarly, other types of nanodevices, microdevices, in-flow devices, and waveguides could be designed [119]. 

By incorporating plasmonic nanomaterials with an exactly controlled shape and size of each of these parts and placed by controlled 2D patterns, it was possible to tune surface plasmon polaritons (SPPs) arising from metal/semiconductor interfaces [120], enhancing light emission in light-emitting devices [121]. Then, the most recent studies on optical surfaces by surface nanopatterning based on plasmonic resonance allowed for controlling the polarization of light [122], which led to on-chip applications based on six metasurface arrays [123]. In addition, interest in these subwavelength polarizations of light will lead to developments associated with the efficiency of organic light-emitting diodes (OLEDs) by controlling the orientation of the transition dipole moment of the emitters and the resonance of the nanoarray resonators [124]. Then, from the combination of semiconductors and plasmonic nanomaterials, studies into luminescence energy transfer, such as ZnO platforms for enhanced directional fluorescence applications, have been conducted [125] and are still in progress.

In addition, research on semiconductors and nanomaterials incorporated in silica waveguides could develop light guides with atomically thin materials for on-chip near-field plasmon detection by semiconductors such as molybdenum disulfide [126]. In this context, the sensitivity of plasmonic waves and the generation of photons from luminescent phenomena are currently being studied. As for example, filtering the time-resolved luminescence signal of quantum optical circuits by single excitation of well-ordered InGaAs quantum dots generated fluorescence resonance at the nanometer scale [127]. Moreover, novel approaches into modified silica waveguides by polymeric stamps were devised to produce several microns in length of one-dimensional nanoparticle assemblies. Combining this technique with the use of tunable, metaldielectric, core-shell nanoparticles [128] allowed obtaining linear assemblies with adjustable inter-particle separation for luminescence propagation applications within resonant plasmonic waveguides [129]. Therefore, the ultraluminescence generation of in silica substrates from MEF for chemical sensing to be applied in on-chip [130] and in-flow applications arouses increasing interest in biosensing [131]. The ultraluminescence properties based on MEF by interaction of the electromagnetic field in the near field of the plasmon with fluorophores can be explained by different pathways such as (i) increased absorption with a higher upper excited state occupation; (ii) decreased non-radiative decays; and (iii) increased radiative decay. Therefore, this effect depends on the distance of the fluorophore from the metallic surface since the electromagnetic field intensity decays exponentially (1/r^6^), drastically affecting fluorophore excitation [132].

To conclude, insights gained from nanomaterials applied to biosensing can give rise to new approaches for targeted studies in NGS, and in this context, it should be highlighted as an example how the development of better control of materials and modified substrates with nanopores has led to their incorporation in real technology on the market. For example, nanopore genome sequencers [133] based on the DNA strands passing through biological pores permitted their identification. The detection was by measuring the difference in their electrical conductivity while the DNA passes through the pores. This level of technology has recently shown interesting applications at the level of rapid identification of pathogens, antibiotic resistance genes, and plasmids in blood cultures in real time [134]. Extracted bacterial DNA of positive blood cultures led to a comprehensive analysis for diagnostic purposes with an acceptable degree of accuracy for these types of samples. Developments from this mentioned study have opened up new possibilities for future applications in clinical microbiology, healthcare surveillance, and precision medicine.

**Figure 15:**
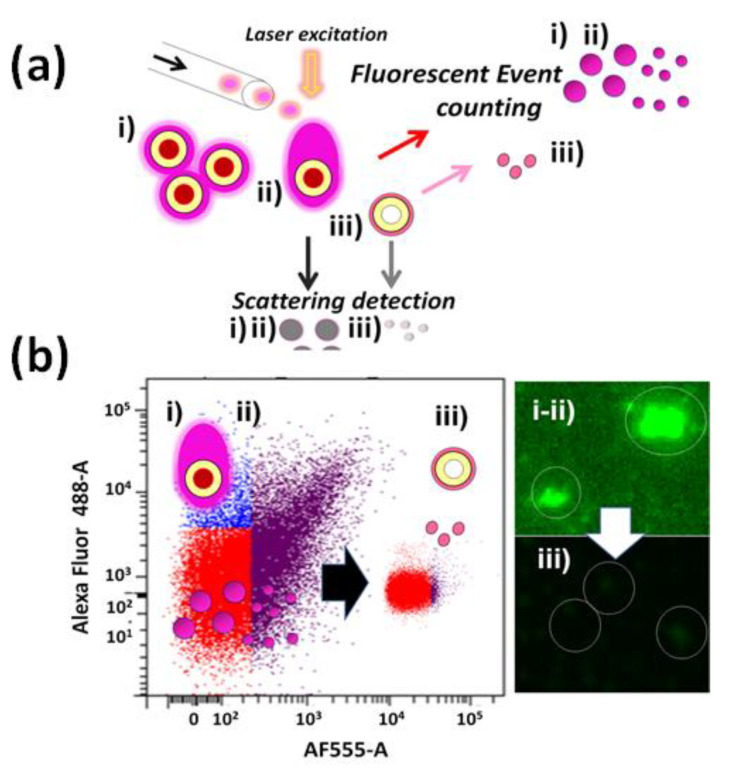
Enhanced nano-emitter detections: (**a**) scheme of in-flow detections of ultraluminescent gold core-shell nanoparticles (Au@SiO2-RhB NPs) and core-less nanoarchitectures ((--)@SiO2-RhB). Distributions of luminescent events of (i) small Au@SiO2-RhB nanoaggregates, (ii) single Au@SiO2-RhB detections, and (iii) core-less (--)@SiO2-RhB were collected, while variable scattered light (i,ii) from core-shell nanoparticles and (iii) from core-less structures was recorded. (**b**) In-flow cytometry by analysis of Alexa Fluor 488-A vs. AF555-A contour plots from core-shell NPs (i) and (ii) to core-less NPs. Inset images (i,ii) corresponded to detection of core-shell NPs and (iii) to core-less NPs by laser fluorescence microscopy. Reprinted with permission from ref. [136]. Copyright 2021, Elsevier.

Finally, in this context, it should be highlighted how nanotechnology is developed within the close relationship between the sizes and physical phenomena involved in the genomic materials, leading to a focus on the nanoscale accompanied by advanced optical setups. Therefore, how the spectroscopy is being developed should be highlighted along with the fact that new spectroscopical techniques are progressively showing insights into biodetection and related research fields and applications, for example, MEF-based strategies, surface enhanced Raman spectroscopy (SERS), and related techniques. In this manner, the engineering of new optical setups is still in progress, improving techniques and methods by incorporating colloidal dispersions of optical active nanomaterials as well as modified substrates to prepare optical active surfaces and porous materials, too (Figure 15) [135]. This concept opens a broad vision to develop microarrays towards nanoarrays and NGS [136]. Thus, it is up to a multidisciplinary research point of view to propose new materials and technology associated with NGS and biosensing. 

## Data Availability

Further results/details can be found by writing to the correspondent author.
